# Factors associated with abandoning exclusive breastfeeding in Mexican mothers at two private hospitals

**DOI:** 10.1186/s13006-020-00316-6

**Published:** 2020-08-19

**Authors:** María Natividad Ávila-Ortiz, Ana Elisa Castro-Sánchez, Elizabeth Andrea Martínez-González, Georgina Mayela Núñez-Rocha, Adriana Zambrano-Moreno

**Affiliations:** Autonomous University of Nuevo Leon, Faculty of Public Health and Nutrition, Calle Eduardo Aguirre Pequeño y Yuriria, s/n Col. Mitras Centro, C.P. 64460 Monterrey, Nuevo León Mexico

**Keywords:** Breastfeeding duration, Exclusive breastfeeding, Risk factors

## Abstract

**Background:**

Exclusive breastfeeding (EBF) has multiple benefits for both the child and the mother; however, there is little data regarding the reason why Mexican mothers with a high socio-economic level abandon EBF before 6 months, and there is limited information about the practice of breastfeeding in private hospitals. The objective was to identify the factors associated with the cessation of exclusive breastfeeding in Mexican mothers at two private hospitals.

**Methods:**

A cross-sectional study was conducted with 218 upper-class mothers selected according to their place of residence by geographic location, socio-economic level, and pediatric consultations cost. They were over 18 years old and with children aged 6 to 24 months.

Data were collected between July and November 2016 by face to face interview using a structured questionnaire while the mothers waited for the pediatric postnatal care consultation in two private hospitals in northeastern Mexico. Exclusive breastfeeding was measured according to World Health Organization (WHO) recommendations, which consist of providing only breast milk for the first 6 months of life. Chi-squared tests and multivariate logistic regression were performed.

**Results:**

Mean maternal age was 31.4 years (SD of 4.4) and most of the participants had an undergraduate education, were married, and worked outside the home. The prevalence of exclusive breastfeeding at 6 months was 28%. Upper-class working mothers are less likely to continue breastfeeding. There was a negative association with employment (AOR 13.69; 95% CI 1.59, 111.11), bottle use in the first 6 months (AOR 7.93; 95% CI 3.07, 20.48), and a low level of knowledge (AOR 2.18; 95% CI 1.04, 4.56). After 6 months, only 61 mothers (28%) maintained exclusive breastfeeding.

**Conclusions:**

Knowledge level, bottle use, and employment are associated with premature cessation of EBF in Mexican upper-class mothers, attending two private hospitals. There was a high percentage of breastfeeding cessation in the sample. It is necessary to reinforce a strategy that coordinates the action of the different laws, regulations and programs affecting the exclusive breastfeeding practice, in order to adequately promote breastfeeding and support mothers in both public and private sectors.

## Background

In 2016, there were approximately 2.7 million deaths globally in children with malnutrition under 5 years of age, a figure that could decrease significantly with the practice of exclusive breastfeeding [1]. Despite the benefits of breastfeeding, mothers often choose not to continue it, and it is estimated that only 38% of children worldwide are exclusively breastfed during the first 6 months of life [2]. In Mexico, according to the National Health and Nutrition Survey (ENSANUT), the prevalence of EBF up to 6 months in 2018 was 28.8% [3]. In 2015, the National Survey of Children and Women (ENIM) reported a figure of 31% [4].

Studies in various countries affirm that factors such as maternal age, education level, employment, health services, immediate attachment, breastfeeding knowledge, problems of the nipples, mastitis, and low milk supply are associated with the practice of exclusive breastfeeding [5, 6]. It has also been observed in different studies that proper counselling, mother friendly work environment and the promotion of EBF are key for increasing the prevalence of mothers practicing exclusive breastfeeding [7, 8].

The research on which this text is based constitutes only the analysis of quantitative data from a mixed EBF study in Mexican upper-class women, who are among the least studied groups. However, there is limited information on the causes of early cessation in upper-class women who attend private hospitals. Most of the studies in this matter are performed in low, middle or disadvantaged economic sectors [9–12]. This creates a gap in knowledge that impedes deep comparative analyses with segregated data between women from different socio-economic levels, identifying their specificities but also making evident common barriers and facilitators. As Lagarde [13] has shown that women and their specific conditions are interrelated. The category “woman” refers to a group of cultural characteristics that women have because of their gender and from real-life conditions, such as access to material and symbolic goods, their type of work, and their conception of motherhood.

On the other hand, it is relevant to emphasize that UNICEF has pointed out that in developing countries, social classes with a greater purchasing power tend to imitate industrialized countries and an increase in mothers who do not want to breastfeed has been observed. Likewise, in countries with low income, women are highly influenced by the nutritional transition and the publicity of the food industry, which exalt the technification of baby food including the use of milk formula and baby bottles as a symbol of the liberated, modern western women with economic success [14]. Paradoxically, EBF in poorer women is higher than in rich families; however, the general trend is a reduction in the practice of breastfeeding.

Women who reside in geographic areas classified in a high level of socio-economic well-being were defined as upper class. It was also determined by national and regional statistics by income level and geographic location, likewise, the costs per consultation and the prices for maternity services were estimated [15, 16].

It is important and necessary to increase the literature and knowledge regarding the EBF women’s conditions and practices, therefore, the objective of this study was to identify the factors associated (Primipara, Type of pregnancy, Mode of delivery, Information on breastfeeding at the hospital, Use of bottle in hospital, Bottle use in the first 6 months, Pacifier use in the first 6 months, Support from partner in breastfeeding, Support of partner in housework, Breastfeeding is pleasant, Cracked nipples, Mastitis, Medication, Milk insufficient, Working Knowledge) with the cessation of EBF in children from six to 24 months of upper-class mothers.

## Methods

### Design

This was a cross-sectional study of potential factors associated with the cessation of exclusive breastfeeding in Mexican mothers, and data were collected by face to face interview using structured questionnaire while the mothers waited for the pediatric postnatal care consultation in two private hospitals in northeastern Mexico. The study was approved by the Ethics Committee of the School of Public Health and Nutrition of the Universidad Autonoma de Nuevo Leon.

#### Setting

The research was conducted between July and November 2016 in the waiting room of the pediatric area of two private hospitals in northeastern Mexico with the collaboration of women with children between six and 24 months of age. In Mexico, the population that attends private hospitals represents 23.1% of the total population, and due to the high costs of consultations, the majority have a high socio-economic level. Nuevo Leon is a border state with the United States of America, it ranks first in childhood obesity worldwide, it is the Mexican state with the greatest urbanization (90%), it has the highest user population of private medical services (16.1%), it also has the highest prevalence of cesarean section births [3, 17]. Two private hospitals with pediatric clinics gave the facilities permission to carry out this study. They are among the most expensive and exclusive maternity hospitals. Its consultation costs varied between 100 and 120 US dollars; and the delivery service costs between 2500 and 3000 US dollars [18]. In addition, these hospitals have the highest medical technology and they are among the top 10 in Latin America [19].

### Sampling and data collection

The sample size was determined using the National Health and Nutrition Survey [8], which reports that the percentage of women that provide EBF is 14.4% at 6 months. The sample was calculated using a formula for the estimation of a proportion with an infinite population, *n* = Z^2^ **p q**/ e^2^, where ***n*** was the sample size, **Z** was the test statistic for the 95% confidence interval, **p** is the population proportion (0.144), and **q** (.856) and **e** were the desired proportion (0.05). After calculation, the minimum sample size for the interviewed mothers was one hundred and eighty-nine.

The respondents were upper-class women, older than 18 years, with children between six and 24 months of age and that they self-reported not having restrictions to breastfeed because of serious or contagious diseases. The women whose children were premature and who had been in neonatal intensive care were excluded. Before applying the survey, all agreed to participate in the study and signed an informed consent form. This was a convenience sample that was integrated consecutively as the mothers who met the inclusion criteria arrived. The survey was then applied and later the children were weighed on calibrated scales by trained nurses.

#### Outcome variables

The outcome variable for this study was duration of EBF since birth, measured according to WHO recommendations, which consist of providing only breast milk during the first 6 months of life, with no other fluids are given, not even water, but the baby could receive oral rehydration salts, drops, and syrups (vitamins, minerals and medication).

To determine the duration of EBF during the study, the mother was asked up to what month of age EBF was provided, up to what month of age only breast milk and water were provided, and up to what month of age semisolid foods were provided. Responses were compared and if the response was greater or equal to 6 months, it was considered EBF and it was recorded as “1”; the mothers that answered less than 6 months or who provided their children with food in addition to breast milk were recorded as “0”. The intention of these questions was to have a more definite answer about the time the baby was breastfed and to ensure that the answers were less influenced by what the mother understands as exclusive breastfeeding.

#### Independent variables

Breastfeeding is influenced by several factors. This study used a questionnaire [20] that explores factors associated with early cessation of exclusive breastfeeding. Independent variables are nominal and were classified into categories: 1) sociodemographic characteristics of the mother (age, marital status, level of education); 2) characteristics before pregnancy and during delivery: primiparous (yes/no), type of pregnancy (planned/unplanned), mode of delivery (vaginal/caesarean section); 3) in-hospital characteristics: EBF information (Did you receive information about breastfeeding during your hospital stay? yes/no), bottle use (During your hospital stay, after delivery, did your child receive a bottle? yes/no); 4) characteristics of the child’s feeding: bottle use (yes/no), pacifier use (yes/no); 5) partner support: decision to breastfeed (yes/no), household tasks (Did you receive support from your partner in housework? yes/no); 6) causes for cessation of EBF self-reported: mastitis (yes/no), cracked nipples (yes/no), use of medications (yes/no), insufficient milk (yes/no), work (yes/no).

For assessing mothers’ knowledge, The Breast Feeding Knowledge Questionnaire was used [21] (Cronbach’s Alpha 0.87). This is a questionnaire based on the WHO and UNICEF breastfeeding recommendations. This instrument has 15 items (e.g. Breastfeeding is the best feeding for the baby; Exclusive breast feeding is recommended for the first 6 months; Breastfed babies are rarely constipated) and each one has a value of 1 point. The scoring range is: 1–5 points is a low level of knowledge; between 6 and 10, a regular level of knowledge; and between 11 and 15 a high or good level of knowledge. Then, the variable knowledge from ordinal to nominal was recoded, grouping low level and regular level as “Low/average”; and high level as a “high” [22].

#### Data analysis

For non-categorical variables, the mean and standard deviation were calculated. For categorical variables, frequencies, percentages, and the 95% confidence intervals were obtained. The association between variables was assessed using the chi-squared test with a *p* < 0.05; the magnitude of the association was determined using an odds ratio. A multivariate analysis with binary logistic regression was performed, where variables with a *p* - value less than or equal to 0.05 (mode of delivery, use of bottle in hospital, bottle use in the first 6 months, support from partner in breastfeeding, milk insufficient, working, knowledge) in the univariate analysis were included in the model. The dependent variable was EBF and the independent variables were: type of pregnancy, method of delivery, primipara, information about breastfeeding in the hospital, use of bottles and pacifiers in the first 6 months, support of the partner to breastfeed and in housework, having cracked nipples, mastitis, taking medication, believing that they did not have enough milk, work, and knowledge about breastfeeding. The statistical program SPSS v 21.0 was used to analyze the data.

## Results

A total of 235 mothers belonging to high-income population were recruited; however, five did not meet the inclusion criteria, four declined to participate and eight had scheduling conflicts. A total of 218 mothers participated and completed the survey (Table 1). The mean age of the children was 13.87 months with a SD of 5.8.

**Table 1:** Sociodemographic characteristics of the participants (*N* = 218)

Characteristic	Exclusive breastfeeding	
	No, ***n*** = 157 (%)	Yes, ***n*** = 61 (%)
Age (years)		
20–29	48 (30.5)	9 (14.7)
30–39	99 (63)	45 (73.7)
40–45	10 (16.3)	7 (11.4)
Marital status		
Married	140 (89.1)	59 (96.7)
Cohabiting	9 (5.7)	0
Single mother	3 (1.9)	2 (3.2)
Divorced/separated	5 (3.1)	0
Education		
Doctorate	3 (1.9)	1 (1.6)
Master’s degree	29 (18.4)	20 (32.7)
Bachelor’s degree	116 (73.8)	38 (62.2)
High school	8 (5.0)	2 (3.2)
Secondary	1 (0.63)	0
Occupation		
Homemaker	75 (47.7)	34 (55.7)
Remunerated job	82 (52.2)	27 (44.2)

**Fig. 1:**
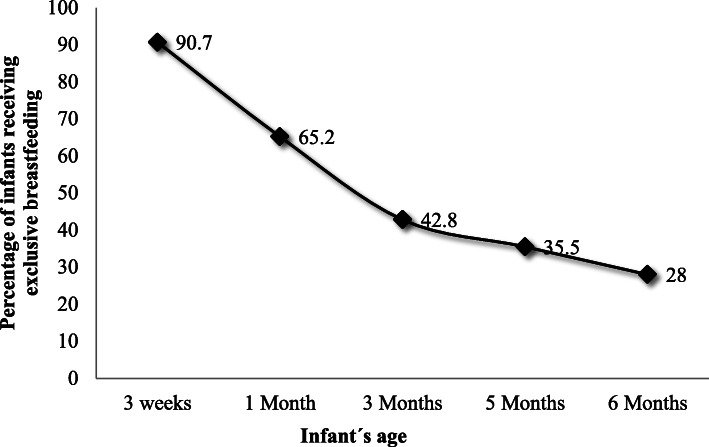
Distribution of rates of exclusive breastfeeding at different ages (*N* = 218)

Of the total mothers, only 28% exclusively breastfed for 6 months, with the majority ceasing exclusive breastfeeding between 3 and 4 months of the child’s life (Fig. 1). Mothers abandoned breastfeeding at 3.3 ± 2.1 months.

In our sample, 48.2% were primiparous, 77.1% of the pregnancies were planned, and 72% were delivered by cesarean section. The use of a bottle during the first 6 months of life predominated in 82.5% as did the use of a pacifier (65.6%). Regarding the support of the partner in breastfeeding, 94% supported their partner and 91.3% helped with housework. Overall, 70.2% of the mothers considered breastfeeding to be pleasant. The main reasons for abandoning exclusive breastfeeding before 6 months were that the mother perceived that she did not produce enough milk (42.9%), followed by the use of medications (17.7%) and employment (16.2%). In addition, 50.5% had a high level of knowledge about breastfeeding (Table 2).

**Table 2:** Bivariate analysis of factors associated with exclusive breastfeeding of infants from 6 to 24 months (*N* = 218)

Variable	Total ***N = 218*** ***n*** (%)	Exclusive breastfeeding		Bivariate	
		No ***N =*** 157 (72%)	Yes ***N =*** 61 (28%)	OR (95% CI)	***p*** - value
Primipara					
Yes	105 (48.1)	75 (71.4)	30 (28.6)	0.94 (0.52,1.70)	0.852
No	113 (51.8)	82 (72.6)	31 (27.4)		
Type of pregnancy					
Planned	168 (77.0)	122 (72.6)	46 (27.4)	1.13 (0.56,2.27)	0.717
Unplanned	50 (22.9)	35 (70)	15 (30)		
Mode of delivery					
Vaginal	61 (27.9)	36 (59)	25 (41)	2.33 (1.24,4.38)	0.008*
Cesarean section	157 (72.0)	121 (77.1)	36 (22.9)		
Information on breastfeeding at the hospital					
Yes	190 (87.1)	137 (72.1)	53 (27.8)	1.03 (0.42,2.49)	0.976
No	28 (12.8)	20 (71.4)	8 (28.5)		
Use of bottle in hospital					
Yes	150 (68.8)	120 (80)	30 (20)	3.35 (1.79,6.24)	0.000**
No	68 (31.19)	37 (63.2)	31 (36.8)		
Bottle use in the first 6 months					
Yes	179 (82.4)	143 (79.9)	36 (20.1)	7.63 (3.56,16.38)	0.000**
No	38 (17.4)	13 (34.2)	25 (65.8)		
Pacifier use in the first 6 months					
Yes	143 (65.5)	105 (73.4)	38 (26.6)	1.22 (0.66,2.26)	0.522
No	75 (34.4)	52 (69.3)	23 (30.7)		
Support from partner in breastfeeding					
Yes	205 (94.0)	144 (70.2)	61 (29.8)	1.42 (1.30,1.55)	0.020*
No	13 (5.9)	13 (100)	0 (0)		
Support of partner in housework					
Yes	199 (91.2)	144 (72.4)	55 (27.6)	1.20 (0.43,3.33)	0.715
No	19 (8.7)	13 (68.4)	6 (31.6)		
Breastfeeding is pleasant					
Yes	153 (70.18)	106 (69.3)	47 (30.7)	0.61 (0.31,1.22)	0.167
No	65 (29.8)	51 (78.5)	14 (21.5)		
Cracked nipples					
Yes	31 (15.6)	23 (74.2)	8 (25.8)	1.00 (0.41,2.40)	0.995
No	167 (84.3)	124 (74.3)	43 (25.7)		
Mastitis					
Yes	15 (7.5)	10 (66.7)	5 (33.3)	0.67 (0.21,2.06)	0.485
No	183 (92.4)	137 (74.9)	46 (25.1)		
Medication					
Yes	35 (17.6)	27 (77.1)	8 (22.9)	1.20 (0.51,2.86)	0.665
No	163 (82.3)	120 (73.6)	43 (26.4)		
Milk insufficient					
Yes	85 (42.9)	56 (65.9)	29 (34.1)	0.46 (0.24,0.89)	0.020*
No	113 (57.0)	91 (80.5)	22 (19.5)		
Working					
Yes	32 (16.1)	31 (96.9)	1 (3.1)	13.36 (1.77,100.6)	0.001**
No	166 (83.3)	116 (69.9)	50 (30.1)		
Knowledge					
Low/average	108 (49.5)	86 (79.6)	22 (20.4)	2.14 (1.16,3.95)	0.013*
High	110 (50.45)	71 (64.5)	39 (35.5)		

*OR* odds ratio, *CI* confidence interval

*p - value < 0.05 (significantly associated)

**p - value < 0.001 (highly significantly associated)

From the comparative analysis (Table 2), some factors associated with the cessation of EBF were identified. It was found that women whose delivery was by cesarean section were more likely to abandon breastfeeding. There was no statistically significant difference in terms of being primiparous, type of pregnancy (planned or unplanned), or receiving information about breastfeeding in the hospital.

Additionally, bottle use at the hospital and during the first 6 months of the child’s life and having a partner who did not support breastfeeding contributed to a significantly higher frequency of EBF cessation compared to mothers who did not use a bottle and felt supported by their partner. The use of a pacifier, support from the partner in housework, and the feeling generated during breastfeeding (*p* > 0.05) were not risk factors for the cessation of breastfeeding (Table 2).

A bivariate analysis was conducted, and the significant variables associated with EBF cessation were: mode of delivery (OR 2.33; 95% CI 0.24, 4.38; *p* = 0.008); mothers thinking that they did not have enough milk (OR 0.46; 95% CI 0.24, 0.89; *p* = 0.020); use of bottle in hospital (OR 3.35; 95% CI 1.79, 6.24; *p* = 0.000); support from partner in breastfeeding (OR 1.42; 95% CI 1.30, 1.55; *p* = 0.020); bottle use in the first 6 months (OR 7.63; 95% CI 3.56,16.38; *p* = 0.000); working (OR 13.36; 95% CI 1.77,100.6; *p* = 0.001) and knowledge (OR 2.14; 95% CI 1.16,3.95; *p* = 0.013) (Table 2).

Multivariate logistic regression analysis included the seven factors that were significant (*p* < 0.05). Four factors were no longer significant (mode of delivery, milk insufficient, support from partner in breastfeeding and use of bottle in hospital), while factors related to EBF cessation at 6 month were the bottle use in the first 6 months (AOR 7.93; 95% CI 3.07, 20.48; *p* = 0.00), work (AOR 13.69, 95% CI 1.59, 111.11; *p* = 0.017), and low knowledge level (AOR 2.18; 95% CI 1.04, 4.56; *p* = 0.037). Therefore, the probability of EBF increases in mothers who do not have paid employment outside the home, when bottle use is minimized during the first 6 months, and when mothers have high knowledge of breastfeeding.

## Discussion

Within the framework of a growing medicalization of births, in Mexico there is a culture of not breastfeeding and giving formula to babies, giving this a high social value as if it were a question of comfort, innovation, and economic success for women and their families [14]. Therefore, it is not a surprise that EBF has decreased; however, there is a lack of data regarding the wealthy population. Highly educated women actively participate in formal employment with economic autonomy, and they are mostly users of private medical services, where limited information regarding breastfeeding is provided [17]. It is relevant since approximately one-fifth of the Mexican population hires private health services and a large percentage of women are attended in private mother-children hospitals; nevertheless, these hospitals adhere less to the Baby-friendly Hospital Initiative (BFHI) [23]. In this context, we must situate the findings of this study as well as those of the UNICEF [23] regarding mothers with a high income who are the ones who mostly decide not to breastfeed and can buy artificial milk that is promoted as preferable to breast milk. Given this, in 2014, in Mexico, the National Breastfeeding Strategy [24] was started, which amplifies the existing conditions so that working mothers can breastfeed their children during work hours, and can have anywhere, free access to milk banks, receive education on the subject, and regulate the marketing of substitutes; however, Mexico continues to be one of the Latin American countries with the lowest rates of exclusive breastfeeding.

This study clarifies and provides knowledge regarding breastfeeding in Mexican upper-class women, as shown by Bueno and Chantry [14], they are considered an ideal referral for wide sectors of women despite the fact that their practices of breastfeeding contradict established international recommendations.

The results of this study open pathways and suggest implications for the current policy of promoting EBF, which somehow intends to restore optimal levels of breastfeeding. A relevant point is to revert the socio structural and cultural factors that influence women so that authorities in the matter can improve the monitoring of compliance with health policies and respect the rights of children in private as well as public hospitals.

One relevant finding is that only 28% of mothers exclusively breastfed their child until 6 months; these figures are below those recommended by the World Health Organization (WHO) [6], and also they are lower than those published by the last National Health and Nutrition Survey 2018 [3] conducted in Mexico and the National Survey of Children and Women [4] conducted in Mexico, too. These low percentages are close to those reported by other authors in Ethiopia [25] who found a prevalence of 26.4%. This is probably due to the similarities between the study participants; both samples came from high-income populations, and most of the surveyed mothers were between 20 and 30 years old. Hence, it is important to further investigate this issue.

Regarding this study, it is important to point out that most of the women were highly educated (16 years) compared to the national average (9.1 years), and 50% of the respondents had paid employment and higher salaries compared to the national average (38%) [17]. In relation to this, UNICEF states that when a woman enters the labor market and does not have legislative support or support from work, her probability of breastfeeding diminishes [23].

This study found that one of the factors for not continuing EBF was paid employment outside the home, a finding similar to a study conducted in Spain [26], where working mothers had a 2.65 times greater risk of early weaning. Other studies have indicated that working mothers are less likely to maintain exclusive breastfeeding until 6 months compared with mothers who do not work [27–31]. For Mexican mothers, returning to work impedes the continuity of breastfeeding, despite the National Breastfeeding Strategy [24] that declares that nursing rooms should be available at their place of work for mothers to express milk or have an hour to breastfeed or manually express milk, however, these actions are not carried out since there is no organization that oversees compliance. In contrast, a study conducted in Cuba [32] indicated that working mothers had higher breastfeeding rates, achieved because the labor legislation in that country monitors compliance, protects motherhood and childhood, and enables women to more easily breastfeed their children.

A strong association between bottle use in the first 6 months of the child’s life and cessation of EBF was identified. This finding is compatible with the results of a study conducted by Camargo et al. [20] in a Colombian population group where not using a bottle in the hospital was the factor most related to a longer duration of exclusive breastfeeding. In this regard, a study carried out in Brazil demonstrated that feeding with a bottle interfered with the orofacial development of the baby, mainly in position and muscular action of the lips and tongue; in addition, position and inadequate suction favor irregular and insufficient milk extraction [33].

Regarding knowledge levels, mothers with a high level of knowledge about breastfeeding were more likely to provide exclusive breastfeeding. Previous studies found that knowledge about breastfeeding influences the prevalence of successful breastfeeding [34–36] since knowledge plays an important role in deciding and making informed decisions regarding exclusively providing breast milk to their child.

In the current study, as in others [5, 32, 37–39], the mothers’ perception that they did not produce enough milk was related to the cessation of breastfeeding. Therefore, the cessation of breastfeeding was found to be related to the perceptions of mothers, potentially, it could be addressed with more information that emphasizes the difficulties that can occur at the beginning of breastfeeding [25]. Perception of low milk supply may be due to lack of confidence in breastfeeding and milk supply, and it needs to be addressed by providing support, education, rather than emphasizing of problems.

A study conducted in the United States [40] found that the main cause of cessation of breastfeeding was problems with the nipples, with a prevalence of 32%. Another study [41] also indicated that mothers who reported problems with the nipples were more likely to abandon EBF early. However, in the current investigation, only 15.2% of the participants abandoned breastfeeding due to issues with the nipples, and this issue did not influence the overall cessation of EBF. This problem can change with orientation and improving breastfeeding technique.

In other investigations carried out in Brazil [42–44], pacifier use was found to be one of the factors associated with EBF interruption. This finding is in contrast to that found in the current study. For the participants, this was not a significant factor for cessation. Also, the American Academy of Pediatrics suggested the use of pacifiers as a way of preventing sudden infant death syndrome, in contrast to recommendations made by the WHO and UNICEF, a situation that can confuse mothers [17].

The current study found an association between the delivery method and exclusive breastfeeding. In contrast, in a study conducted in Medellin, Colombia [45], in a population similar to this one, there was no such association. Similar findings have been reported by Ahmed y Salih [46] who pointed out that cesarean section was a risk factor for starting breastfeeding. In this regard, the ENSANUT [3] points out that one out of every two cases are a cesarean section and that in the private sector they comprise almost 70% of births. In addition, a mother and her child are separated for several hours almost immediately after birth, a period during which other liquids are offered to the newborn. Among these, formula, promoting the use of breast milk substitutes and generating unsuccessful breast milk production, are all in contradiction to what the WHO proposes.

The support of the partner was found to be a protective factor for EBF, this agrees with findings from a study carried out in Bogotá, Colombia [47] that reports that the partner is a very important source of emotional support that positively stimulates, gives confidence and the will to prolong breastfeeding. Fathers that have greater participation in prenatal care favor breast milk as a way of feeding the child and they are more active in the process of helping and motivating their partner [48]. Other research has indicated that parental support can influence decisions and behavior regarding breastfeeding. Likewise, the mothers’ perception of their partners’ responsiveness and the parents’ reports on their own responsiveness predicted a greater intent and a longer duration of breastfeeding [49].

The results of this study cannot be generalized to women of different socio-economic sectors because the participants belonged to a high-income group and they were only from northeastern Mexican region bordering the United States of North America. The use of convenience sampling method interfered with the representativeness of the collected data. In addition, the duration of EBF that was reported could be a limitation. However, to reduce this bias, women with children aged 24 months or younger were surveyed and they were asked about the age at which they started feeding formula and the age at which they stopped in order to obtain reliable information.

## Conclusion

In conclusion, it was found that the prevalence of EBF in mothers with a high income in Mexico is lower than in populations from other Latin American countries. The study found that EBF drops sharply after 3 months of the child’s life and an important association was documented between bottle use in the first 6 months, employment status, and breastfeeding knowledge. After these, the other factors with a statistically significant relationship with EBF were the mother’s perception of not producing enough milk, the method of delivery, and bottle feeding during the hospital stay.

Breastfeeding after childbirth was low among Mexican women of high socio-economic levels. Future studies should consider to examine the influence of not having partner support along with that cesarean births increase the chances for abandoning exclusive breastfeeding. In addition, private hospitals should be monitored to implement the National Breastfeeding Strategy as well as UNICEF’s international initiatives on this issue. It is of the utmost importance that national policy makers consider successful intervention evidences based on the women’s particular characteristics for designing breastfeeding programs. It is also necessary to continue with this research line in populations with different marginalization degrees, and strengthen the legal labor framework to promote and protect the practice of exclusive breastfeeding.

## Data Availability

Data used in this study are available upon reasonable request. Please contact the corresponding author for data requests.

## Abbreviations

AOR: Adjusted Odds Ratio
BFHI: Baby-friendly Hospital Initiative
CI: Confidence Interval
EBF: Exclusive Breastfeeding
ENSANUT: Encuesta Nacional de Salud y Nutrición [National Health and Nutrition Survey]
ENIM: Encuesta Nacional de los Niños, Niñas y Mujeres [National Survey of Children and Women]
INEGI: Instituto Nacional de Estadística y Geografía [National Institute of Statistics and Geography]
OR: Odds Ratio
UNICEF: United Nations Children’s Fund
WHO: World Health Organization
